# Barriers and facilitators of road traffic injuries prevention in Iran: a qualitative study 

**Published:** 2019-08

**Authors:** Naser Derakhshani, Ghader Darghahi, Hojat Gharaee, Shabnam Vahedi, Nima Pour-golam

**Affiliations:** ^*a*^Student Research Committee, Iran University of Medical Sciences, Tehran, Iran.; ^*b*^Road Traffic Injury Research Center, Tabriz University of Medical Sciences, Tabriz, Iran.; ^*c*^Tabriz Health Services Management Research Center, Health Management and Safety Promotion Research Institute, Tabriz University of Medical Sciences, Tabriz, Iran.

## Abstract

**Background:**

Identifying the barriers to and facilitators of the prevention of traffic injuries from the ‎perspective of the key stakeholders and experts seems indispensable. Objectives: The present ‎study aims to identify the barriers to and facilitators of the prevention of traffic injuries in Iran. ‎

**Methods:**

In this qualitative study with conventional Content-Analysis approach, 42 key stakeholders and ‎experts in the field of traffic injuries in Iran were selected based on purpose and theoretical ‎sampling to reach informational saturation and their views concerning barriers to and ‎facilitators of the prevention of traffic injuries in Iran were studied using semi-structured ‎interviews. Data was analyzed using Content-Analysis method. ‎

**Results:**

Five main barriers including: structural barriers, organizational barriers and planning, socio-‎cultural barriers, scientific barriers and intersectional barriers and 22 sub-themes were ‎extracted. The lack of lead agency, which was among structural barriers, was selected as the ‎main barrier. In addition, five general facilitators including sensitization of society and ‎authorities, use modern of facilities and equipment, increase of attention to safety (vehicles and ‎roads), increase of information and awareness, use of expert manpower were extracted. The ‎sensitization of society and authorities was selected as the most important facilitator. ‎

**Conclusions:**

From key expert’s viewpoint, the barriers to the prevention of traffic injuries are more than their ‎facilitators. Therefore, planning and paying more attention to removing these barriers and ‎promoting the facilitators seems necessary to reduce traffic Injuries. Select a lead agency with ‎adequate powers and resources can be the highest priority of intervention.‎

**Keywords:**

Road traffic injuries, Prevention, Barriers, Facilitators

